# Design and evaluation of the “Feel Good” feasibility study – a multi-component fruit and vegetable intervention in children measuring cognitive and mental health outcomes

**DOI:** 10.1186/s12937-025-01137-1

**Published:** 2025-05-14

**Authors:** Nicola A. Gillies, Jeanette P. Rapson, Amy L. Lovell, Karen E. Waldie, Clare R. Wall

**Affiliations:** 1 Department of Nutrition and Dietetics, Faculty of Medical and Health Sciences, University of Auckland, Auckland, New Zealand; 2 School of Psychology, Faculty of Science, University of Auckland, Auckland, New Zealand

**Keywords:** Fruit, Vegetables, Behaviour change, Flanker task, Executive functioning, Randomised controlled trial, Children, Cognition, Mental health, Behaviour

## Abstract

**Background:**

Observational evidence suggests that increasing fruit and vegetable (FV) intake has the potential to improve children’s cognitive function and mental well-being, but this has not yet been empirically tested in intervention research. This study assessed the feasibility and acceptability of a multi-component FV intervention which measures mental and cognitive health outcomes in children.

**Methods:**

The ‘Feel Good Study’ was a cluster-randomised controlled feasibility study conducted in four New Zealand primary schools, with equal allocation of schools to intervention and wait-list control arms. The intervention group received a 10-week FV programme informed by behavioural theory, including school- and home-based components designed to improve FV availability and acceptance. The wait-list control group received a simplified 5-week version of the intervention. Dietary, cognitive, and mental health outcomes were completed by children and parents/caregivers at the start and end of the 10-week study period. Primary outcomes of this feasibility study were recruitment, retention, and data collection rates. Process evaluation captured measures of intervention fidelity and dose, acceptability, reach, and barriers or facilitators to implementation.

**Results:**

Seventy children were recruited (79% of target recruitment rate), with an average retention rate of 89%. Diet, cognitive, and mental health data collection procedures were feasible, with all data valid for analysis except for 6% of children’s dietary questionnaires. All intervention components were delivered (100% dose delivered), with high levels of fidelity (82% - 100% of components implemented as planned). All teachers and parents strongly agreed that they would recommend other schools/families take part in the study, indicating high levels of acceptability. Process evaluation revealed areas for refinement including more cohesive connections between school- and home-based intervention components, strengthening or adding new intervention components, and simplifying enrolment procedures with longer recruitment periods.

**Conclusion:**

Having satisfied key feasibility and acceptance measures in the Feel Good Study, we recommend intervention refinement and progression to a definitive trial where the efficacy of increased FV intake for mental health and cognitive function can be tested in children for the first time.

**Trial registration:**

The trial protocol was prospectively registered with the Australia and New Zealand Clinical Trials Registry (ACTRN12623000533695) on 2 May 2023, https://www.anzctr.org.au/Trial/Registration/TrialReview.aspx?id=385829&isReview=true.

**Supplementary Information:**

The online version contains supplementary material available at 10.1186/s12937-025-01137-1.

## Background

Eating a variety of fruits and vegetables (FV) throughout childhood provides essential nutrients for growth, development, and overall health [1–3]. Healthy dietary patterns, including those high in FV intake, established in childhood are likely to track into adulthood, decreasing chronic disease risk [4, 5]. Yet many children world-wide continue to fall short of recommended intakes of FV. In Aotearoa New Zealand, only 5% of children aged 5 to 14 years eat enough vegetables daily, while 73% eat enough fruit [6], which aligns with evidence globally [7, 8]. These trends persist across genders and ethnicities, but fewer children in the most deprived areas meet fruit recommendations compared to those in the least deprived regions [6].

Beyond just physical health, growing observational evidence suggests that greater FV intake can enhance aspects of children’s mental health and cognitive functioning [9–12], supporting their overall life achievements [13]. Rising childhood obesity rates [6, 14] has long-been a global imperative for investment in strategies to ensure adequate FV intake in children [15–17], yet increasing rates of mental health concerns adds weight and urgency to this issue [18, 19].

Schools are a promising setting for interventions seeking to increase FV intake. Schools not only have the potential to reach high numbers of participants, but also allow for a more equitable environment to influence broad determinants of FV consumption, and allow for positive reinforcement from teachers and peers [20]. This is true for scalable public health interventions, but also interventions to evaluate the impact of increased intakes on health outcomes such as cognitive functioning or mental well-being.

Multi-component approaches targeting both school and home environments show greater potential for increasing children’s FV intake, and therefore would allow for the effects of FV on mental health or cognitive function to be examined [20]. While a number of approaches can be used to increase fruit intake through school-based interventions, increasing vegetable intake has proven more challenging [21]. A meta-analysis of primary school healthy eating programs underscores the effectiveness of experiential sensory learning strategies (i.e. learning through doing and using the five senses: taste, sight, hearing, smell and touch) [22]. For instance, a recent Australian program integrated these methods into a vegetable-specific education initiative, resulting in increased vegetable knowledge, acceptance, and willingness to eat them, overcoming challenges encountered by previous school-based strategies [23].

Here we evaluate a multi-component FV intervention utilising sensory learning methodologies, while addressing evidence gaps (i.e. the effects of increased FV on child mental wellbeing) [24]. Following the Medical Research Council guidance on complex interventions [25], we report the design, feasibility testing, and process evaluation of a multi-component intervention designed to increase FV intake and measure cognitive and mental health outcomes in children. Specifically, the primary objective was to determine the feasibility of recruitment, retention, and data collection measures of the Feel Good Study. A secondary objective was to evaluate the acceptability and fidelity of intervention implementation and research procedures. This paper offers a comprehensive overview of the study protocol and reports findings from the process evaluation. Outcome measures concerning dietary behaviours, mental health, and cognition and power calculations for a definitive trial will be addressed in a separate publication.

## Methods

### Study design and setting

The Feel Good Study was a 10-week wait-list controlled, cluster-randomized feasibility study conducted in primary schools located in Auckland, New Zealand. For the purpose of this feasibility study, four classrooms were invited to participate. The four classrooms were recruited from four different primary schools to allow for a spread of sociodemographic characteristics. Recruitment and randomization procedures took place between July – September 2023. Data was collected at the start and end of school term 4 of 2023 (10 weeks, October – December). The active intervention arm received their 10-week intervention during school term 4 of 2023, while the wait-list control received their simplified 5-week intervention at the start of term 1 of 2024. No formal power calculation was completed. A sample size of 70 participants has been recommended for feasibility trials in order to estimate sample sizes required for a fully-powered RCT [26].

**Fig. 1:**
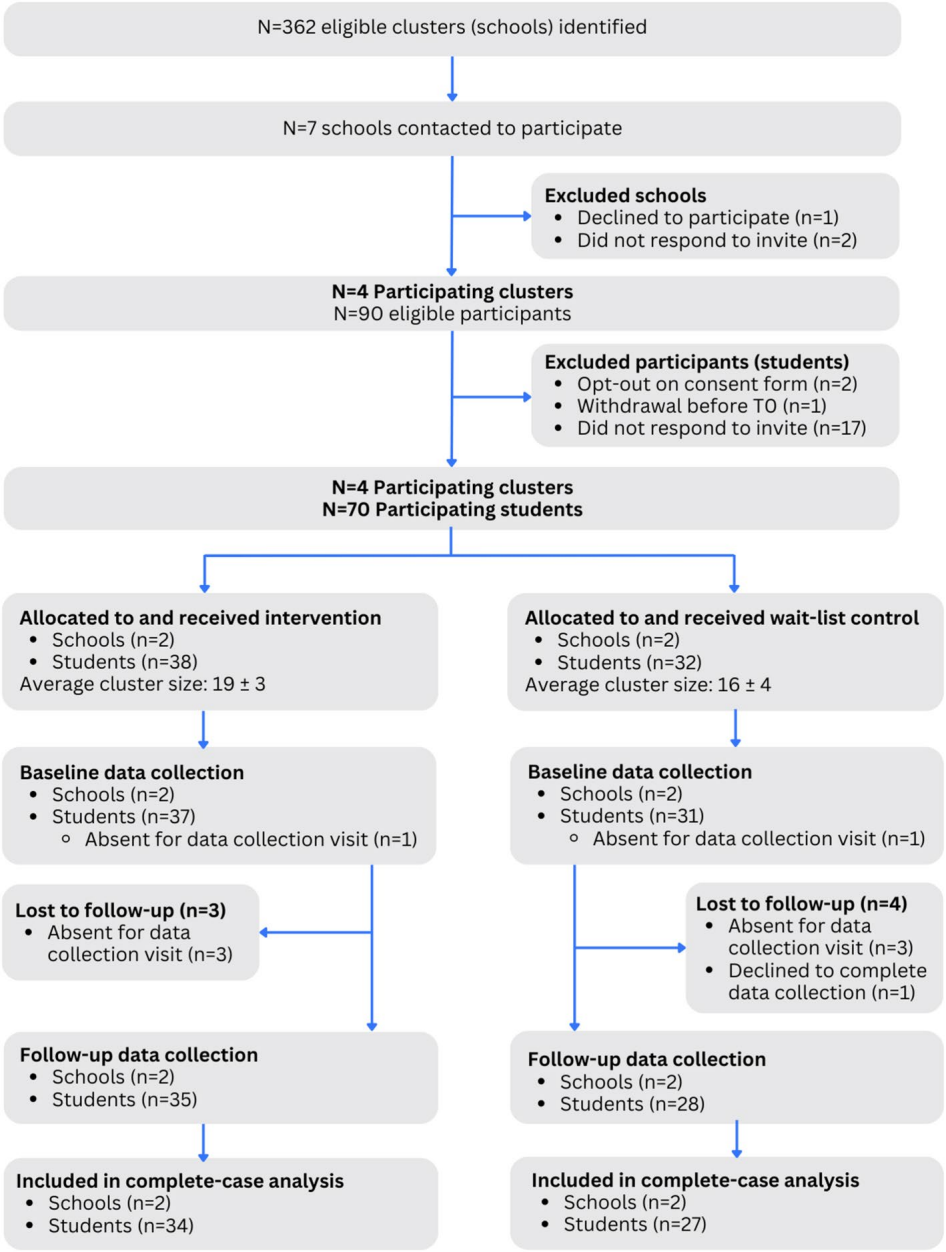
CONSORT diagram showing participant flow through the Feel Good Study

Ethical approval for the study was obtained from the National Health and Disability Ethics Committee (2023 EXP 18255), and the study was pre-registered with the Australian New Zealand Clinical Trials Registry (ACTRN12623000533695). The protocol reported here was in accordance with the SPIRIT (Standard Protocol Items: Recommendations for Interventional Trials) and CONSORT guidelines for pilot and feasibility studies [27]. The CONSORT extension for pilot and feasibility trials flow diagram is shown in Fig. 1, and a CONSORT checklist can be found in Supplementary File 2.

### Participants

#### Inclusion and exclusion criteria

The main study population were children in school years 4-6 (aged 7-11 years) attending primary school in Auckland, New Zealand. Data was also collected from parents/caregivers (herein referred to as parents), teachers and principals, who can also be considered as participants.

Children would be excluded from data collection if they were unable to complete cognitive and psychosocial assessments (e.g., autism spectrum disorder requiring significant support with regards to communication or completing the assessments, unable to speak/understand English). The decision to exclude participants was guided by a developmental psychologist in the research team (KEW) in conversation with the child’s parent/caregiver.

#### Informed consent

Informed consent was first required from the school principal, a Board of Trustees representative, and a classroom teacher for the classroom to take part in the study, including for researchers to deliver school-based intervention components to the classroom. Dual consent (parents) and assent (children) was required for each child to participate fully in the study, including data collection and home- and school-based intervention components. Participation in the study was entirely voluntary and in compliance with the International Conference on Harmonization (ICH) guidelines.

Recognising that there are many reasons why parents are not able to sign consent forms (e.g., low levels of literacy, limited time), all children in the participating classroom could take part in the school-based intervention components independent of parent signed consent, but would still not participate in any data collection without completed consent and assent forms. Parents or children could opt out of participation in classroom activities if they wished in their returned consent or assent forms, which meant that these children would not participate in classroom activities or data collection. This opt-out process was used with the intent to minimize feelings of exclusion for children in participating classrooms.

### Recruitment and stratification

Schools were stratified into tertile bands according to the New Zealand Equity Index (EQI), a 226-point numerical scale from 344 – 569, with greater EQI representing greater socioeconomic barriers to achievement at school [28]. The intention for this feasibility study was to recruit two schools each from the upper and lower EQI tertile, given that socioeconomic status potentially has a moderating effect on feasibility (e.g., recruitment) or other outcome (e.g., scope for change in FV intake) measures. Due to limited engagement from high EQI (greater socioeconomic barriers) schools within the time constraints of recruitment for this feasibility study from initial email and phone invitations, the protocol was adjusted during recruitment to recruit schools in the lowest (high socioeconomic status (SES)) and upper-middle EQI (lower SES) bands.

Principals were first approached for participation in the study via email invitations using opportunistic sampling (i.e., approaching schools based on existing relationships and networks, or information provided by the Ministry of Health School Directory). Following a series of introductory meetings with principals and teachers, children were invited to take part in the study via a classroom visit and parents through a visually informative email invitation letter. Due to only needing four schools for this feasibility study, invitation emails were sent to one school at a time. If schools did not respond to our contact efforts within two weeks, an invitation to another school was sent. As shown in Fig. 1, a total of seven schools were contacted before out target of four schools was reached, with one school declining the invitation to participate and two schools not responding to the invitation email.

### Randomisation and allocation

Following full consent, classrooms matched for EQI were randomised on a 1:1 allocation. Randomisation sequences were prepared by a researcher not involved in recruitment or data collection (ALL), and managed centrally through REDCap (Research Electronic Data Capture [29]) during the trial. Participants were not informed of the intervention allocation before consent, though due to the need for adequate time for planning, teachers were informed of their classroom’s allocation by one researcher (JR) before some assent/consent forms had been returned from children and parents. This was an unblinded trial due to the nature of the intervention, and participants were also not blinded to study hypotheses.

### Intervention

The intervention was developed in accordance with the Medical Research Council’s guidance on complex interventions [30] and by Registered Dietitians in the research team, in collaboration/consultation with key community stakeholders (teachers/principals, Māori (indigenous to New Zealand) and Pacific health professionals, sensory education specialists). Stakeholders were invited to review our intervention plan and resources both via email and in a meeting format to encourage discussion. Key feedback incorporated into the final intervention was centered around strategies to promote cultural responsiveness for Māori and Pacific families (e.g., karakia (blessing) before any food is consumed, avoiding food waste, avoiding food touching the head), and intervention components or resources that could be added (e.g., the garden bucket, recommendations for resources from community organizations). The TIDieR checklist can be found in Supplementary file 3.

The intervention was designed as a behaviour change intervention, using the behaviour change wheel theoretical framework. A COM-B (capability, opportunity, motivation) behavioural analysis was undertaken, followed by identifying intervention components and implementation options using a standardised behaviour change technique taxonomy [31]. A full description of this process is described in Supplementary file 1.

In brief, the multi-component intervention included home- and school-based strategies, combining FV delivery to overcome barriers relating to availability, alongside a behaviour change support package to enhance acceptance through building capability and motivation.

#### Intervention components

The full 10-week intervention programme was developed to align with the timeframes of school terms in New Zealand. The 10-week intervention was used for the intervention group, while a simplified 5-week version of the intervention was used for the wait-list control group in this feasibility study in the interest of time and logistical constraints. A summary of school- and home-base support intervention components are summarised below, and in Table 1.

**Table 1:**
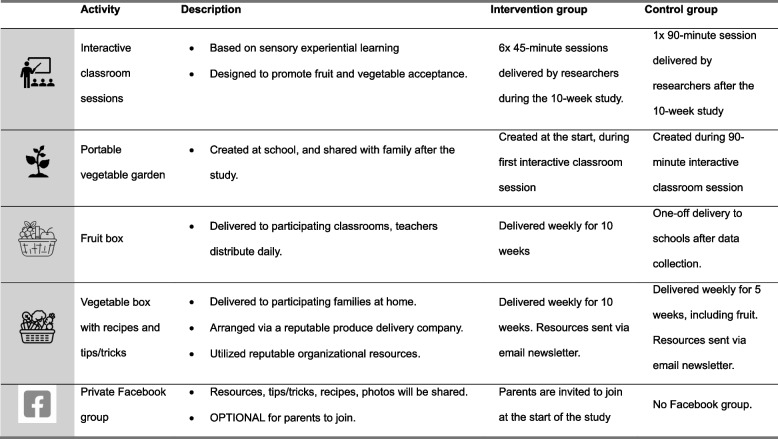
Summary of school- and home-based intervention strategies utilized in the Feel Good Study for intervention and wait-list control groups

##### School-based components (intervention group)

Participating classrooms received a weekly fruit box delivery for the duration of the 10-week intervention, with flexibility for the classrooms to decide how this was best implemented.

**Fig. 2:**
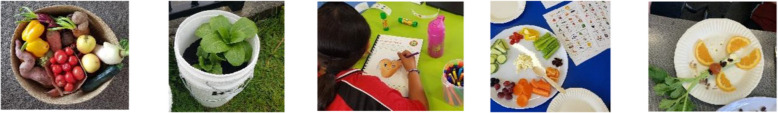
Photographs taken during the delivery of sensory experiential learning lessons, including (from left to right): perfect vs imperfect vegetable exploration, portable garden, lesson workbooks, sensory platters and fruit and vegetable bug activity

Research dietitians (NG and JR) delivered six lessons in the classroom, including an introductory/gardening session and five sensory experiential learning lessons. The gardening session involved growing a transportable garden in a bucket from vegetable or herb seedlings, which they grew together over the course of the 10-week study and then took them home. Sensory experiential learning was integrated into school lesson plans, using the Flavour School programme and Sapere approach as a guide, which encourage children to explore food through sight, smell, touch, hearing and taste, fostering curiosity and acceptance of a variety of foods [32]. Workbooks, teaching manuals, and other supporting materials for the implementation of sensory experiential learning were specifically designed for this study (JR), which included integration of contingent reinforcement through role modelling, sticker rewards, and activities (Fig. 2). An overview of the lesson plans is provided in Supplementary table 3.

##### Home-based components (intervention group)

Participating families received a weekly vegetable box delivered to their homes, accompanied by recipes that utilised the vegetables and other tips and tricks (e.g., strategies to help children eat well, tips to reduce food waste) delivered via weekly email newsletters. Existing reputable resources were utilized for the recipes (e.g., Heart Foundation, Vegetables NZ), which provide recipe and skill development cards for a range of cultures, and skill levels in New Zealand.

**Fig. 3:**
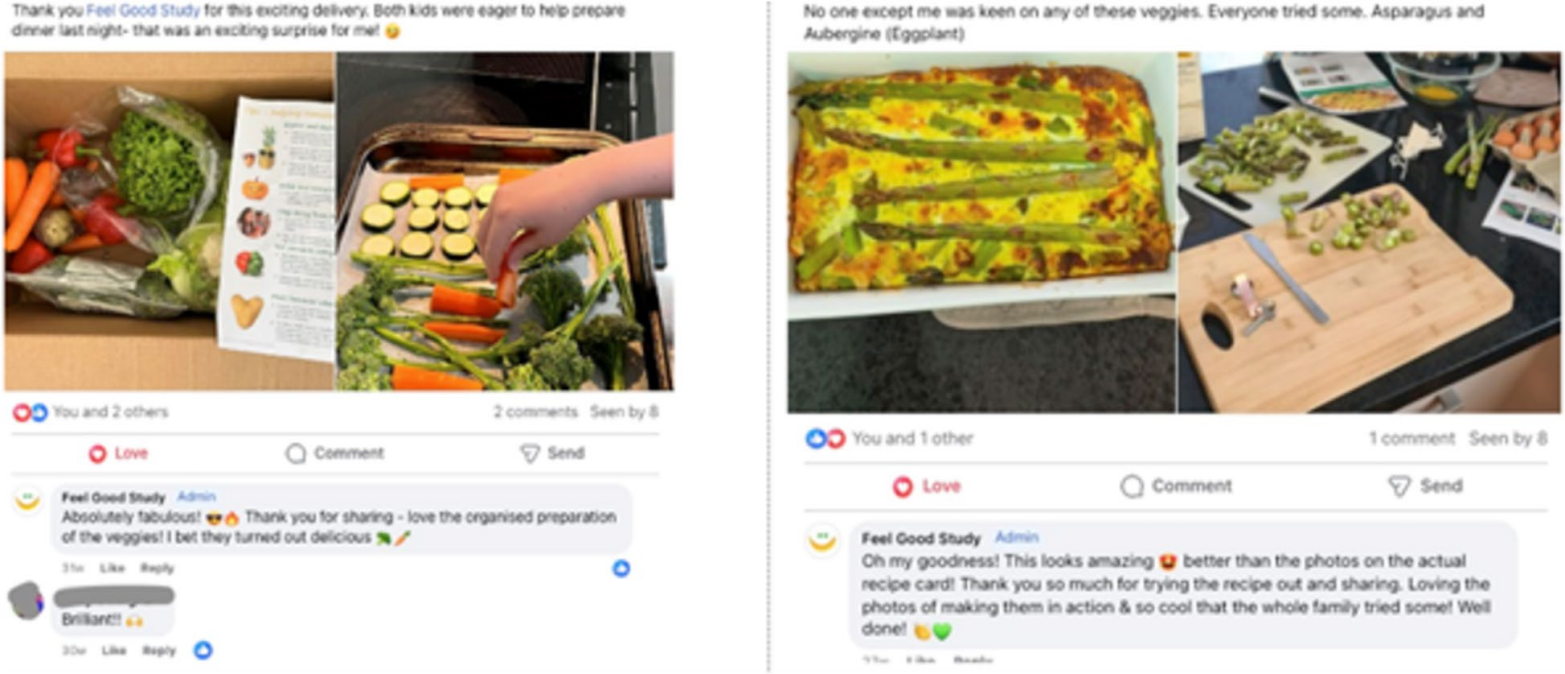
Selection of posts from Facebook group, demonstrating the vegetable box delivery with resources, and interaction between participants and researchers

Parents were invited to join a private study Facebook group with other participants from within the same classroom. The Facebook group was intended to promote engagement with the researchers and other participants of the study, including the classroom teachers (Fig. 3). Parents were invited to take part in a weekly photo challenge through the Facebook group and weekly newsletter, where each photo posted or submitted using produce from the weekly box was considered an ‘entry’ to a random prize draw at the end of the study.

##### Wait-list control components

The wait-list control received a simplified 5-week version of the intervention described above. This included 5-weeks of fruit and vegetable box deliveries to participating families, with accompanying recipes, tips and tricks, and the invitation to take part in the photo challenge in a weekly email-newsletter. Wait-list control classrooms received a 90-minute combined gardening and sensory experiential learning lesson, and a one-off fruit box delivery after data collection was completed. The structure of the simplified intervention was driven by scheduling constraints with the study being conducted in term 4 of the school year.

### Data collection and measurements

Data were collected at baseline and the10-week endpoint from participating children in classrooms by two trained researchers (JR, NG). Children first completed paper-based questionnaires, with staff present to assist with completion. Children then completed Veggie Meter® assessments in the classroom, before completing the NIH toolbox® cognitive assessment. Parents and teachers completed online surveys via REDCap [29], with reminders sent over email and text message if not completed within two days.

#### Feasibility measures (primary outcome)

A summary of measures to assess trial feasibility are summarised in Table 2, which includes data from both research records and process evaluation. Pre-defined targets for recruitment, retention, and data collection were set at 80% for this feasibility study to proceed without modification to a definitive, fully-powered RCT in the future.

**Table 2:** Outcomes, definitions, and data sources used to assess trial feasibility

**Outcome**	**Definition**	**Data source**
Recruitment	% of enrolled participants (children) from those that were eligible and invited to participate.	Recruitment records kept by researchers in excel files and REDCap, class rolls.
Retention	% of enrolled participants (children) completing follow-up data collection (questionnaires)	Data collection records kept by researchers in REDCap
Data collection and analysis	% of enrolled participants (children) completing questionnaires and cognitive assessment which were valid for analysis	Data collection records kept by researchers in REDCap.
Biological sample collection^a^	% of participants (children) opting in for stool sample collection, and % completing baseline and follow-up sample collection.	Recruitment and data collection records kept by researchers in REDCap.
Intervention implementation	Dose delivered and fidelity of study processes and intervention as planned.	Process evaluation tools – Core components checklist.
Intervention acceptability	Acceptance and satisfaction of study processes and intervention.	Process evaluation tools – End of lesson (children) or intervention (parents, teachers) surveys.

^a^See Supplementary file 1 for details regarding at home-collection protocols

#### Diet, cognitive, and mental health measures

Dietary, cognitive, and mental health measures were collected at baseline and week-10 from children and parents. Changes in these measures according to intervention group are not presented in the current manuscript, and will be described in detail elsewhere including their selection and adaptation to the study context. The overview presented in Table 3 is intended to give context to the scope of measures collected.

**Table 3:** Overview of diet, cognitive, and mental health measures utilised in the Feel Good Study

**Outcome**	**Participant**	**Measurement tool**
Baseline data collection^a^		
Health and demographics	Parent	Purpose-designed survey
School environment	Teacher	Purpose-designed survey
Child’s diet		
FV, beverage, and snack intake	Children	Components from the NZ children’s FFQ [33]
FV acceptance	Children	5-point hedonic facial scale [23]
Willingness to try FV	Children	Yes/no question item [23]
Diet quality	Parent	DICE questionnaire [34]
Food neophobia	Parent	Food neophobia scale [35]
Skin carotenoids^b^	Children	Veggie Meter® [36]
Child’s mental health		
Positive Affect	Children	PROMIS positive affect scale [37]
Behaviour	Parent	Strengths and Difficulties Questionnaire [38]
Child’s cognitive function^c^		
Executive function	Children	NIH toolbox – Flanker test
Processing speed	Children	NIH toolbox – Pattern comparison test
Language	Children	NIH toolbox – Picture vocabulary test

All tools except for purpose-designed surveys have been validated for use in children of this age group, or have been adapted from those used previously in similar research in this age group (FV intake, FV acceptance, willingness to try FV)

*Abbreviations*: *DICE* Dietary Index of a Child’s Eating, *FFQ* food frequency questionnaire, *FV* fruit and vegetable

^a^All other measures were collected at baseline and the 10-week follow-up

^b^The Veggie Meter® is a non-invasive, painless fingertip device used to measure skin carotenoid levels

^c^Completed with two students at a time in a quiet room, which took approximately 15-minutes to complete on an iPad

#### Process evaluation

The guide from Saunders et al. [39] informed our process evaluation plan, selected for its specificity to health promotion interventions. Key elements of the Feel Good Study process evaluation included recruitment, implementation fidelity (quality of implementation), dose delivered (extent of intervention implementation), dose received (satisfaction among children, parents, and teachers), reach of the intervention into the child population, and context (barriers or facilitators to implementation). Researchers, teachers, parents, and children were all involved in the process evaluation.

Process evaluation data was used for both formative and summative purposes in this study. Formative data included immediate feedback (e.g., emails and text messages), which could be used to fine-tune the program as it was running or keep the program on track. Key process evaluation instruments include a core components checklist completed by researchers, end of intervention surveys completed by parents and teachers with qualitative ((1) What did you like most about this study? (2) What are some things you would like to see changed? and (3) Is there anything else you would like to tell us?) and quantitative responses, and brief hedonic facial enjoyment and satisfaction scales completed by children at the end of each lesson. A detailed summary is provided in Supplementary file 1.

### Participant retention

To enhance recruitment and retention, schools received a sports voucher at the end of the study, and teachers received a gift. Children received a surprise gift voucher at the end of the study, which they were not aware of on the recommendations of the ethics committee.

### Data analysis

Feasibility and process evaluation data analyses were exploratory, and not intended to test the effectiveness of the intervention. Descriptive statistics are presented as numbers (%) of participants for categorical data or mean ± standard deviation for continuous data.

Thematic analysis was used to assess qualitative data from parent and teacher feedback surveys. An inductive approach was taken, with one researcher (JR) generating codes from responses to three key questions: 1) “What did you enjoy most about the study”; 2) “What are some things that you would like to see changed?” and; 3) “Is there anything else that you would like to tell us”. Codes were then organised into potential themes which were reviewed and finalised in collaboration with a second researcher (NG). Representative participant quotes were selected to illustrate key themes.

## Results

### Participant characteristics

Seventy children from four classes participated, with 65 (93%) parents providing baseline demographic information (Table 4). The children’s average age was 10.0 ± 1.1 years, of which 36 (55%) were girls. Nearly half of the participants were NZ European (46%), with low rates of Māori (3%) enrolment.

**Table 4:** Characteristics of participants taking part in the Feel Good feasibility study

	**Total** **(*n*=70)**	**Intervention** **(*n*=38)**	**Control (*n*=32)**
Questionnaires completed	65	35 (92)	30 (94)
Child’s age	10.0 ±1.1	10.3 ± 0.6	10.0 ± 1.1
Child’s gender - Female	36 (55)	20 (57)	16 (53)
Child’s ethnicity^a^			
Māori	2 (3)	-	2 (7)
NZ European	30 (46)	18 (60)	12 (40)
Samoan	2 (3)	2 (6)	-
Tongan	2 (3)	-	2 (7)
Cook Island Māori	2 (3)	-	2 (7)
Niuean	1 (2)	-	1 (3)
Chinese	7 (11)	1 (3)	6 (20)
Indian	11 (17)	7 (20)	4 (13)
Other – Asian/Middle Eastern	13 (20)	7 (20)	6 (20)
Other – European/Latin American	4 (6)	3 (9)	1 (3)
Child’s medical conditions^b^			
Asthma	6 (9)	4 (11)	2 (7)
Allergies or hay fever	4 (6)	3 (9)	1 (3)
Type 1 Diabetes	1 (2)	1 (3)	-
Epilepsy	1 (2)	-	1 (3)
Parent’s relationship to child			
Mother	48 (74)	25 (71)	23 (77)
Father	15 (23)	8 (23)	7 (23)
Other family member	2 (3)	2 (6)	-

Data presented as mean ± standard deviation (continuous variables), or n (%) (categorical variables)

^a^Counts refer to all options chosen, such that one child can have multiple ethnicities recorded

^b^Grouped according to self-reported conditions, only 1 parent indicated they would prefer not to disclose

### Recruitment, retention and data collection (primary outcomes)

Four of the seven schools contacted took part in the Feel Good Study, and the study was able to recruit 71 (79%) of the 90 eligible students (Table 5). There were some differences in enrolment rates between high (low EQI tertile) and lower (upper-middle EQI tertile) SES groups, with greater participant enrolment rates for schools in high SES areas (92% – 100%) compared to lower SES areas (60% – 64%). Retention rates were good in both intervention (81% – 96%) and control (85% – 92%) groups. There were high data collection completion rates for the total sample at baseline (>90%) and follow-up (>80%), with minimal attrition across time (Supplementary table 7). Only 8 (6%) of children’s dietary questionnaires were excluded from further analyses due to validity concerns, including incomplete questionnaires with >25% of missing items (*n*=6) or major inconsistencies between baseline/follow-up data which indicated children’s lack of understanding of the questionnaire (*n*=2). Full parent/child consent was low for optional stool sample collection (13% of total study participants), as was collection rates, with only 4 (44%) and 2 (22%) children completing collection at baseline follow-up, respectively (Supplementary Table 8).

**Table 5:** Summary of participant recruitment, enrolment, and retention

	**Total**	**Intervention**		**Control**	
		High socioeconomic status	Lower socioeconomic status	High socioeconomic status	Lower socioeconomic status
Class size	90	25	25	20	20
Recruitment					
Parent/caregiver EOI	74 (82)	23 (92)	19 (76)	20 (100)	12 (60)
Parent/caregiver consent	71 (79)	23 (92)	16 (64)	20 (100)	12 (60)
Child assent	77 (86)	23 (92)	19 (76)	20 (100)	15 (75)
Non-participation	2 (2)	0 (0)	2 (8)	0 (0)	0 (0)
Enrolled	71 (79)	23 (92)	16 (64)	20 (100)	12 (60)
Retention					
Withdrawals	1 (1)	1 (4)^a^	0 (0)	0 (0)	0 (0)
Lost to follow-up^b^	7 (10)	0 (0)	3 (19)	3 (15)	1 (8)
Completion rate	63 (89)	22 (96)	13 (81)	17 (85)	11 (92)

Values are displayed as n (%), referring to the proportion of eligible students from the classroom (recruitment), or proportion of enrolled participants (retention)

^a^Participant moved schools after enrolment, but before data collection started

^b^Refers to children who did not complete data collection (questionnaires) at follow-up

### Fidelity & dose delivered

Implementation was primarily assessed through the “core components” checklist completed at the end of the intervention, which captures both study processes and elements implemented in the FV intervention. Overall, our findings show strong agreement that both study processes and intervention elements were implemented as planned in both intervention and control groups (Supplementary table 9).

All study processes and intervention elements included in the core components checklist were implemented across the 4 schools (100% dose delivered). Fidelity scores were more favourable in the control than intervention group, with 100% of home- and 95% of school-based intervention elements implemented as planned compared to 82% of home- and 85% of school-based intervention elements in the intervention group. All data collection processes were implemented as planned (100% fidelity).

Sixteen parents/caregivers joined the Facebook group of the 27 that were invited to join. There were 107 posts, 100 comments and 298 reactions recorded in the Facebook groups combined (Supplementary figure 2).

#### Dose received (enjoyment and satisfaction)

##### Children

Most children in both intervention and control groups responded positively to the lessons according to the brief hedonic satisfaction and enjoyment scales completed at the end of each lesson, with 88-100% of children strongly agreeing or agreeing with the statement “I had fun in today’s lesson” across the different lessons. Average scores ranged from 4.4 out a possible 5 (Primer lesson) to 4.8 (Touch lesson) in the intervention group, and 4.9 for the control group’s ‘Mega Lesson’ (Supplementary Table 10).

#### Parents & teachers

##### Quantitative responses

Most parents and teachers expressed a high level of satisfaction with study processes and intervention elements. Of the 84% of intervention group parents completing the end of intervention survey, 97% either strongly agreed or agreed that they enjoyed taking part in the study, 100% reported that their child enjoyed taking part in the study, and that they would recommend other families to take part in the study. This is comparable to the 100% of parents in the control group who enjoyed taking part in the study and reported that their child did too, with 95% reporting that they would recommend other families take part (Table 6). Parent responses to changes in behaviour because of taking part in the study (Supplementary Table 11) indicated that behaviour change techniques focused on exposure, modelling of the behaviour, and restructuring the physical or social environment were implemented in the home environment.

**Table 6:** Parent -reported feedback in end of intervention survey

	Intervention group, *n*=32 (84% response rate)			Control group, *n*=21 (66% response rate)		
	Very satisfied/satisfied	Neutral	Very dissatisfied/ dissatisfied	Very satisfied/satisfied	Neutral	Very dissatisfied/ dissatisfied
Intervention components						
Veggie Box - Quality	29 (91)	3 (9)	-	20 (95)	-	1 (5)
Veggie Box - Variety	31 (97)	-	1 (3)	21 (100)	-	-
Veggie Box - Amount	31 (97)	-	1 (3)	20 (95)	1 (5)	-
Recipes and information to help families eat more fruit and vegetables	22 (69)	8 (25)	2 (6)	21 (100)	-	-
Enjoyed receiving the weekly veggie box	31 (97)	1 (3)	-	21 (100)	-	-
Portable vegetable garden^a^	25 (78)	2 (6)	3 (9)	15 (71)	1 (5)	-
Study participation						
I enjoyed taking part in the study	31 (97)	1 (3)	-	21 (100)	-	-
My child enjoyed taking part in the study	32 (100)	-	-	21 (100)	-	-
I would recommend other families to take part in the study^b^	32 (100)	-	-	20 (95)	-	-
Research processes						
Opportunity to provide feedback during the study	28 (87)	3 (9)	1 (3)	21 (100)	-	-
It was easy to take part in the study	30 (94)	2 (6)	-	21 (100)	-	-
It was easy to talk with and contact the researchers^c^	25 (78)	6 (19)	-	18 (86)	1 (5)	-
I was well informed about what taking part would involve	31 (97)	1 (3)	-	21 (100)	-	-

Data is presented as n (%)

^a^6% of the intervention group and 24% of the control group responded “not applicable to me”

^b^5% of the control group responded “I would prefer not to answer”

^c^3% of the intervention group and 5% of the control group responded “not applicable to me”

All four teachers strongly agreed that their classroom enjoyed taking part, and they would recommend other schools to take part in the study (Supplementary Table 12).

##### Qualitative responses

A total of 34 parents/caregivers (intervention, *n*=22; control, *n*=12) and all four teachers provided qualitative data. As shown in Table 7, three main themes emerged in response to being asked what they enjoyed most about taking part in the Feel Good Study. Responses highlighted the study’s positive impact—specifically, improvements in children’s willingness to try fruits and vegetables, receiving fresh produce deliveries, and/or their appreciation for the opportunity to participate in a program supporting children’s healthy eating.

**Table 7:** Summary of thematic analysis from parent and teacher responses to the question “what did you enjoy most about the study” in the end of intervention survey

**Theme**	**Responses**	**Illustrative quote**^**a**^
Promoting child food acceptance	Participants valued the study’s impact on their child’s willingness to try different fruits and vegetables	*“[I enjoyed most] that my child is more open to eating a variety of vegetables, and willing to try new ones.” (parent)*
Ease of access and availability	Participants valued the convenience of receiving a free weekly box of fresh fruits and vegetables	*“[I enjoyed most] getting fresh fruit and vegetables delivered.” (parent)*
Gratitude for the Feel Good Study	Participants expressed gratitude for the opportunity to take part in the study, as well as the value it had for improving children’s healthy eating habits	*“Thank you for the experience. We are very grateful for the fruit and vegetables we received. It certainly uplifted attitudinal changes in cooking, eating more fruit and vegetables...” (Teacher)*

^a^See Supplementary table 12 for a comprehensive summary of qualitative feedback

When asked about study improvements, the majority (*n*=23; 68%) of parents/caregivers did not have any suggestions. Of the twelve who provided suggestions, there was a desire for more recipes and healthy eating resources, including fruit in the home deliveries for the intervention group, or wanting more opportunities for child/parent involvement. Teachers from both intervention schools noted concerns around a limited variety of fruit delivered, and suggested additional activities such as using school kitchen facilities to cook together and create a cookbook compiled by students (Supplementary Table 13).

### Reach

The target attendance rate for sensory lessons was 80%, which was met for both the control (84%) and intervention (average attendance 87%) group, although attendance ranged from 66% to 95% across the six lessons for the intervention group (Table 8). The low attendance in lessons 3 (41%) and 4 (64%) for one school was related to a clash with the lessons and a sporting event that researchers were not aware of. Only 17 children (45%) in the intervention group attended the primer and all five sensory lessons.

**Table 8:** Enrolled participant attendance rates across intervention group lessons

Lesson	School A attendance	School B attendance
Primer	88%	95%
Lesson 1 – Sight	94%	95%
Lesson 2 – Smell	88%	91%
Lesson 3 – Touch	100%	41%
Lesson 4 - Hearing	88%	64%
Lesson 5 - Taste	94%	91%

### Adverse events

No adverse events were reported to researchers throughout or at the end of the intervention.

## Discussion

The aim of this study was to examine the feasibility of conducting a cluster-RCT which seeks to increase FV intake in children and measure cognitive and mental health outcomes. The findings demonstrate that research processes are feasible, with targets for recruitment, retention, and data collection rates met alongside evidence that the intervention was implemented with good fidelity. The school- and home-based intervention components were acceptable to children, parents, and teachers with high levels of satisfaction and enjoyment reported throughout and at the end of the study.

The Feel Good Study recruited and retained 100% of schools at the cluster-level, with average recruitment and retention rates at the individual participant level of 79% and 89%, respectively. Recruitment rates varied from 60-100%, with higher recruitment rates observed in schools facing low socioeconomic barriers. This discrepancy in recruitment rates is an issue that needs to be addressed before scaling this intervention into a fully-powered RCT, for example through an internal pilot integrated within a larger-scale RCT to refine recruitment and intervention procedures so that they are acceptable for a diverse population. It is encouraging that we retained those allocated to the control group, as this has been an issue previously reported in dietary interventions delivered in the school setting [23]. Although information sheets and recruitment discussions described our randomisation process, control group teachers did express disappointment in not being allocated to the intervention group. This has the potential to risk compliance and retention, indicating the opportunity to further develop processes such that the intervention and control groups feel that they are getting equal value, or to better manage expectations.

Successful recruitment strategies implemented in this study could be harnessed for a definitive trial. We observed that enthusiastic teachers facilitated excellent participant recruitment, for example through proactively setting targets for children to gain class points if they returned their completed assent form. Researchers fostered this enthusiasm by including teachers in classroom activities (vegetable deliveries, Facebook group, veggie meter measurements) such that a strong, reciprocal relationship was developed. Barriers to recruitment included an initially complicated consent process, which first required parents to submit an expression of interest before then moving onto consent procedures. Previous school-based research in NZ has attributed low participation rates (48%) to requirements for younger children (5y) to sign their own assent forms [40]. Although children in this study were older (7-11y), we found no issues with the use of assent forms, which were age-appropriate and visually appealing. The complexity of information in the parent information sheets and consent form may have also contributed to lower recruitment rates in schools facing greater socioeconomic barriers, similar to observations made elsewhere [23].

The process evaluation shows that the intervention was implemented as planned, with higher fidelity rates than other school-based interventions [41]. Researchers being responsible for the delivery of most intervention items likely explains the high rates of implementation (82-100% fidelity, 100% dose), rather than relying on schools/teachers. Having researchers deliver intervention components is shown to be more effective than teachers [20], and also helps to overcome barriers such as teachers prioritizing the implementation of less time-consuming activities [42, 43]. At the same time, the resource burden of this approach does create concerns for scalability into a definitive intervention. Interestingly, teachers did express willingness to deliver the sensory lessons and a hybrid approach with teachers and researchers could be an appropriate path forward to maintain a high degree of intervention fidelity and low levels of participant burden. The intervention group did have lower rates of intervention fidelity overall, which is likely explained through timing of intervention delivery (lessons learned from the intervention group by the time of control group delivery), or by the more simplified version of the intervention component (e.g., reward stickers provided on just one occasion for the control group, rather than at six occasions for the intervention group).

The most consistent feedback for improvements to the Feel Good Study from parents was a desire for more recipes and healthy eating resources, aligning with the low uptake of this behaviour change technique. Parents also expressed an interest in greater continuity between home and school activities, with opportunity for greater parental involvement. Evaluation of other school-based health promotion programs have similarly resulted in recommendations to encourage parents into schools for intervention components to deepen connections and create a ‘buzz’ to enhance behaviour change [44]. It appears that school-based interventions directly targeting parents (e.g. attendance at sessions) are more likely to lead to improved dietary behaviours in children [45], and one teacher’s recommendations for integrating cooking classes present an opportunity to bridge this gap. Although there was good engagement with the Facebook group for those that signed up, there was limited accessibility to the page as a whole which may have contributed to the disconnect felt by parents overall. An engaging newsletter co-created with students could be delivered instead of the Facebook group and email.

This feasibility study should be interpreted in the context of its strengths and limitations. Process evaluation ensures intervention transparency and informs decisions for further implementation. Embedding formative and summative mixed-method process evaluation components throughout the intervention enabled identification of successful elements, and also helped guide changes needed for a definitive RCT. This is not only relevant for scaling the current study, but others seeking to conduct either FV interventions in children, or behavioural interventions in the nutritional psychiatry or developmental fields. The latter is particularly important, given that there are an increasing number of dietary interventions seeking to improve mental well-being (albeit in adults), yet limited adoption of behavioural frameworks in intervention design [46] or reporting [47]. Process evaluation also serves to ensure that participants feel heard and have the chance to share their experiences, demonstrating a respect for participants’ dignity, well-being, and autonomy. In the context of New Zealand, this also demonstrates a commitment to Te Tiriti o Waitangi through acknowledging participants as valued partners, not merely subjects of a study. The qualitative aspect of our process evaluation could have been further enriched with semi-structured interviews or focus groups at the end of intervention. This was part of our protocol, but was not feasible within the time constraints of the project and conducting the intervention in the final term of the year. Collecting qualitative feedback from children would also add richness to this data, particularly as they are the focus for behaviour change and data collection. Finally, directly asking children, parents, and teachers about uptake of specific behaviour change techniques or incorporating ethnographic practices to have objective data of behaviour change technique use would have better allowed us to optimize intervention content for a definitive trial. As argued by Hankonen et al. [48], even in interventions delivered with high fidelity, if participants do not take up the intended behaviour change techniques then the intervention may still fail to have effect.

The use of a wait-list control was also a strength, and likely contributes to the high levels of acceptance and retention of the control arm, even despite using a simplified version of the intervention. However, closer efforts must be made to reduce expectation effects in a definitive trial, as we did not conceal the purpose or hypothesis of the study during recruitment and informal feedback from the control group during the study was that participants had reported making changes to behaviours around FV before receiving the intervention. We acknowledge the limitations of using opportunity sampling to expedite recruitment in this feasibility study, and a resulting limited understanding of intervention feasibility/acceptability in some population groups. In particular, we were not able to recruit a diversity of schools with respect to socioeconomic status, and we also have low representation of Māori (indigenous population of New Zealand) despite efforts taken with consultation during study design.

Informed by process evaluation of the Feel Good Study, proposed refinements for research processes and intervention elements in a definitive trial include improved study timing, simplifying consent procedures, further development of intervention components, long-term behaviour change evaluation, and remedying the issue of representation in the current feasibility study (Table 9).

**Table 9:** Recommendations for study refinement

	Proposed refinements	Proposed outcome
Participant diversity	Remedy issue of poor representation. An internal pilot within the definitive trial could be considered	Understand feasibility and acceptability to sociodemographic groups not represented in feasibility study.
Study timing	Recruit schools and teachers in the school year before participant recruitment begins.	Time to allow for connections between researchers and participants, facilitating recruitment
Consent procedure	Remove expression of interest ‘gate keeper’ step, and simplify information sheets whilst balancing ethical review board requirements.	Facilitates recruitment from a more diverse sociodemographic background.
Intervention components	Build greater connection between school and home activities	Parents feel more integrated within the intervention programme, facilitating behaviour change uptake.
Follow-up period	Evaluate any lasting effects of the intervention on behaviour change at longer follow-up periods.	Supports evaluation of the programme as a whole, and ongoing funding and research in this area.

## Conclusion

We have shown that it is possible to conduct a multi-component intervention delivered through the school and home environment to evaluate the effects of increased FV consumption in mental and cognitive health in children – a critical, yet under-looked age group with respect to these outcomes. The Feel Good Study was able to recruit, retain, and analyse data from a sufficient number of participants, with study procedures and intervention components acceptable to participants. This warrants progression to a definitive trial with protocol refinements informed by our process evaluation.

## Supplementary Information

Supplementary Material 1.

Supplementary Material 2.

Supplementary Material 3.

## Data Availability

The datasets used and/or analysed during the current study will be made publicly at the University of Auckland figshare repository (https://auckland.figshare.com/) once corresponding outcome data has been published. Until publicly available, data will be made available from the corresponding author on reasonable request.

## Abbreviations

EQI: New Zealand equity index
FV: Fruit and vegetable
SES: Socioeconomic status
