# Supplementary material for: Design and evaluation of the “Feel Good” feasibility study – a multi-component fruit and vegetable intervention in children measuring cognitive and mental health outcomes
Source: Nutr J. 2025 May 14;24:80. doi: 10.1186/s12937-025-01137-1 (PMC12076836; doi:10.1186/s12937-025-01137-1)
Supplement: Supplementary file 1 — Supplementary Material 1. [file 12937_2025_1137_MOESM1_ESM.docx]

# Intervention design and components

The Behaviour Change Wheel (1) integrates the COM-B (capability, opportunity, motivation) behavioural analysis alongside identification of behaviour change techniques using a standardized taxonomy (2). Using this process and taxonomy ensures that interventions are underpinned by psychological theories that link actions and outcomes, and allows for later evaluation of intervention components.

The behaviour change wheel is organized into 3 stages with 8 steps, with details of how the behaviour change wheel was used to guide the design of the Feel Good Study dietary intervention described below.

### Stage 1 – Understand the target behaviour and identify what needs to change.

The target behaviour for the Feel Good Study was adhering to increased fruit and vegetable (FV) intake for the duration of the intervention. A COM-B behavioural analysis provides insight into three components (Capability, Opportunity, Motivation) which play a role in producing and changing the target behaviour. Determinants of children’s behaviours around FV intake are well characterised. Availability or accessibility of FV and taste preferences/lack of neophobia are consistently noted as strongest determinants of FV intake in children (3,4), outlined in **Supplementary Table 1** below alongside other contributing determinants from international and local evidence (3–6).

**Supplementary Table 1. Key determinants of fruit and vegetable (FV) intake in children**

| Personal determinants | Environmental determinants |
| --- | --- |
| - Taste preferences | - Availability and accessibility |
| - Nutrition-related knowledge | - Peer influence |
| - Skills | - Culture |
| - Self-efficacy (Perceived confidence in ability to engage in a behaviour) | - Parental behaviour   - FV intake/modelling   - Structured mealtimes   - Family mealtimes and eating behaviours   - Availability of FV at home   - Parents providing FV to children. |
| - Eating behaviours |  |
| - Interest in a healthy diet |  |
| - Outcome expectations (Perceived positive consequences) |  |

Although knowledge and outcome expectations are identified as determinants of FV intake, the importance of promoting FV as fun, enjoyable, and satiating rather than just healthy is emphasized in the literature. Cognitive reasoning that FV should be consumed because of health benefits should be avoided as this has been shown to undermine acceptance (7).

### Stage 2 – Identify intervention options.

Seven of the 9 intervention functions in the BCW were considered relevant to the current intervention, including Training, Education, Environmental Restructuring, Modelling, Persuasion (using communication to induce positive or negative feelings or stimulate action, e.g., using imagery to motivate increases in FV intake), and Incentivisation.

#### Stage 3 – Identify intervention content and implementation options.

Intervention components are identified through selecting the most appropriate behaviour change techniques. The behaviour change technique taxonomy (BCTTv1) was compared to suggested intervention components, revealing 15 behaviour change techniques that were most likely to bring about behaviour change and 10 were used.

These behaviour change techniques were then mapped onto the Theoretical Domains Framework identified from the COM-B analysis in stage 1 and the 7 selected intervention functions in Stage 2 (**Supplementary Table 2**), and the resulting logic model is shown in **Supplementary Figure 1**.

| Feel Good study logic model | | | | |
| --- | --- | --- | --- | --- |
| 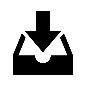 | 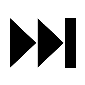 | 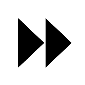 | 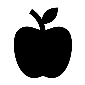 | 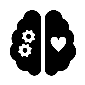 |
| *Inputs* | ***Immediate impacts*** | ***Short-term impacts*** | ***Behavioural impacts*** | ***Health outcomes*** |
| Providing FV to children (via school and home) alongside education (gardening, sensory lessons, recipes) will… | …result in the development of a dietary FV intervention which will… | …result in changes in the school and home environment (i.e., increased availability or access to FV and education, which… | …will result in increased FV acceptance and intake and ultimately… | …may lead to improved mental health and cognitive function in children. |

**Supplementary Figure 1. Logic model for Feel Good Study fruit and vegetable (FV) intervention.**

### Intervention design summary

The behaviour change framework identified key determinants of FV intake in children as acceptance and availability/accessibility. Successful elements from previous research to enhance FV acceptance were integrated into this intervention, including sensory experiential learning elements (8–11), role modelling, repeated exposure, and rewards (12). Increased availability of FV is embedded in the intervention through delivery of fruit and vegetables for the duration of the study period. These intervention components incorporate key behaviour change techniques of role modelling (peer, parents), reward, repeated exposure, and changing the physical and social environment.

**Supplementary Table 2. Links between the COM-B analysis, theoretical domains framework (TDF), intervention functions and behaviour change techniques (BCTs) used to guide the design of the Feel Good Study multi-component dietary intervention.**

| **Source of behaviour (COM-B)** | **TDF** | **Intervention function** | **Potential Intervention components** | **Corresponding BCTs** |
| --- | --- | --- | --- | --- |
| Physical capability   - Skills to prepare food - Skills to cook food | - Skills | - Training | - Cooking classes (school or community) - **Gardening (school, home or community)** | **4.1 Instruction on how to perform a behaviour**  **6.1 Demonstration of the behaviour** |
| Psychological capability   - Knowledge - Self-efficacy | - Knowledge - Beliefs about capabilities | - Education | - **School curriculum components** - **School and home activities** - **Parent education packages** | **7.7 Exposure**  2.4 Self-monitoring of behaviour  1.1 Goal setting (behaviour)  1.3 Goal setting (outcomes)  13.5 Identity associated with changed behaviour  **4.1 Instruction on how to perform a behaviour** |
| Physical opportunity   - Cost of FV - Availability of FV - Time/convenience - School food environment | - Environmental context and resources | - Training - Environmental restructuring | - **FV delivery** - **Parent education packages** | **12.1 Restructuring the physical environment** |
| Social opportunity   - Social norms (peers, community, family) - Modelling (peers, community, family) - Parental behaviour | - Knowledge - Skills - Social influences | - Education - Training - Modelling | - **Parent education packages** - Family/community fun nights/weekend - Having a project buddy (social support) | **12.2 Restructuring the social environment**  **3.2 Social support (general)**  **6.1 Modelling of the behaviour** |
| Reflective motivation   - Outcome expectations (belief around consequences or outcomes) - Self-efficacy - Values around health/wellbeing | - Beliefs about capabilities - Optimism - Knowledge - Intention - Goals | - Education - Modelling - Enablement - Persuasion - Training - Incentivisation | - **School curriculum components** - **Reward system** - Self-monitoring - Goal setting/action plans | **10.1 Incentive**  **10.2 Material reward**  2.4 Self-monitoring of behaviour |
| Automatic motivation   - Taste preferences - Habits | - Memory, attention, and decision processes - Behavioural regulation - Reinforcement | - Training - Enablement - Education - Environmental restructuring - Incentivisation | - **Curriculum components** - **Self-monitoring** - Review goal setting - **Reward system** | **10.1 Incentive**  **10.2 Material reward**  **8.3 Habit formation**  **7.7 Exposure**  **4.1 Instruction on how to perform a behaviour**  **7.1 Prompts/cues** |

Intervention components in bold selected for final intervention, based on feasibility/appropriateness for the intervention setting and study context.

**Supplementary Table 3. Outline of sensory experiential learning sessions included in the school support package for intervention and control groups**

| Lesson/Week | Objectives | Activities | Materials | Examples^1^ |
| --- | --- | --- | --- | --- |
| Primer session 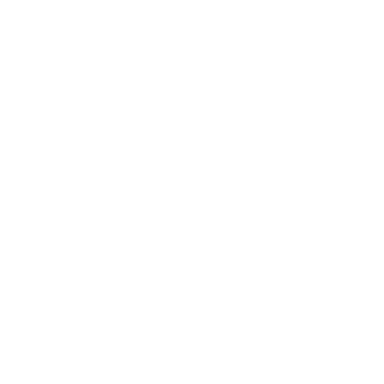 | - Children are introduced to sensory aspects of eating FV through the programme “Teach Your Monster - Adventurous Eater” - Children start a sustainable portable vegetable garden | - Introduction - Gardening - *Teach Your Monster game^2^*   Total time: 60 minutes | - Two buckets per child (one with drilled holes in the bottom) - Soil/seedling/seeds - Garden tools - Computer or smart device | 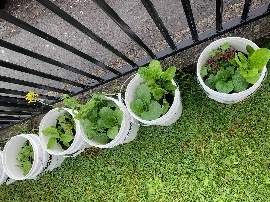Portable gardens |
| 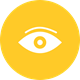1 - Sight | - Children explore kai visually - Children learn about where FV come from. - Children learn and use new vocabulary to describe sensory experiences. - Children learn about how the sense of sight can affect the experience of eating FV. | - Introduction - Compare a kete (basket) of “perfect” vs “ugly” FV - Blind taste/smell test - Design a FV monster in workbook - Evaluation + award sticker   Total time: 45 minutes | - Sight icon - Box of “perfect” FV - Box of “ugly” FV - Magnifying glasses - Blind folds - Monster Workbook - Art materials - Word bank - Stickers | 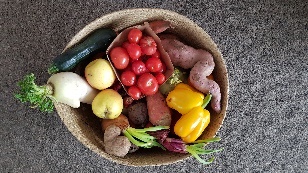“Ugly” vegetables  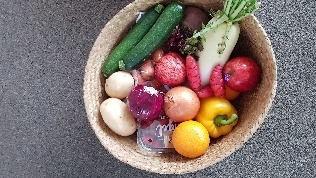  “Perfect” vegetables |
| 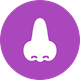2 - Smell | - Children learn how smell can interact to create flavour. - Children become aware of individual differences in FV preferences. | - Introduction - Mystery smell jars - Explore table: smell master - Monster workbook - Evaluation + award sticker   Total time: 45 minutes | - Smell icon - Opaque jars with strong smelling FV, herbs or spices - FV explore tables - Monster Workbook - Word bank - Stickers | 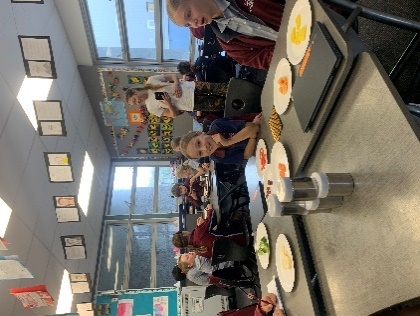  Mystery smell jars & explore table |
| 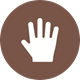3 - Touch | - Children explore FV with the sense of touch. - Children think about how the sense of touch informs their eating experience. | - Introduction - Explore table - Monster workbook - Evaluation + award sticker   Total time: 45 minutes | - Touch icon - FV explore platters - Monster Workbook - Word bank - Stickers | 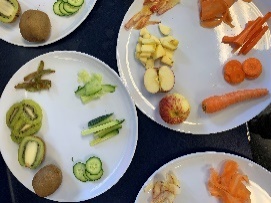  Explore table |
| 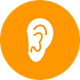4 - Hearing | - Children explore FV with hearing. - Children think about how sense of touch/sound informs their eating experience. | - Introduction - Explore table - FV bugs - Monster workbook - Evaluation + award sticker   Total time: 45 minutes | - Hearing icon - FV explore tables - Cut FV, cream cheese & hummus - Monster Workbook - Word bank - Stickers | 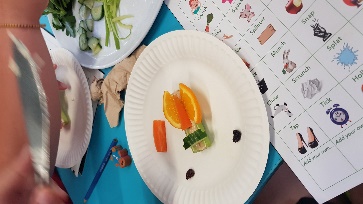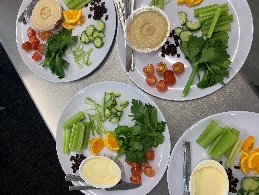Example of FV bug materials and word bank |
| 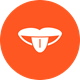5 - Taste | - Children learn the 5 basic tastes and how these inform their eating experience. - Children learn how taste combinations can interact to create flavour. - Children become aware of individual differences in fruit and vegetable preference. | - Introduction - The 5 basic tastes - Taste explorer table: Taste combinations - Finish your monster workbook - Evaluation + award certificates   Total time: 45 minutes | - Taste icon - FV five basic taste plates - FV explore tables - Cut FV + herbs/spices - Monster Workbook - Word bank - Taste Education teaching resource - Stickers & certificates | 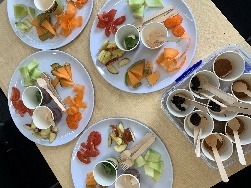  Taste combination explore table |
| Sensory lesson for control group  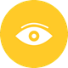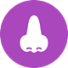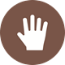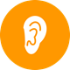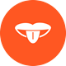 | - Children are introduced to sensory aspects of eating FV - Children start a sustainable portable veggie garden | - Introduction - Gardening - Explore table - FV bugs - Sensory worksheet - Evaluation + award sticker/certificates   Total time: 90 minutes | - Two cups per child (one with drilled holes in the bottom) - Soil/seedling/seeds - Garden tools - FV explore platters - Word bank - Stickers & certificates | 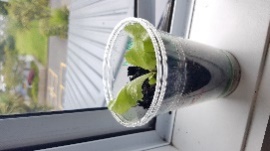  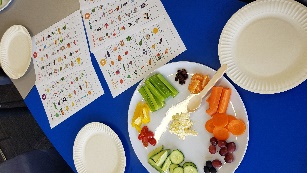  Mini portable garden and flavour explore table |

Abbreviations; FV, fruit and vegetable. ^1^Examples are photos taken during the delivery of sensory experiential learning lessons. ^2^The “Teach your Monster: Adventurous Eating” game is an app- and web-based game, which in the context of this intervention will help introduce children to sensory explorations as a primer before children are provided actual samples of fruits and vegetables to see, smell, touch, hear, and taste during the lessons. Given time constraints, we were unable to play the free web-based version in the classroom with the children in the classroom as intended, and instead decided to send instructions home to parents/caregivers to allow children to play the game at home, similar to the control group.

## Process evaluation methodology

As recommended by Saunders *et al* (13), the development of this methodology has been an iterative and collaborative, incorporating review from the research team and an independent advisor with expertise in evaluation interventions through a health behaviour change lens. The Feel Good Study process evaluation plan was developed to review both study processes (e.g., data collection) and intervention elements (e.g., school- and home-based intervention components). Each process evaluation component was assigned a process evaluation question to ensure focus, and the evaluation's aims and objectives were met (**Supplementary Table 4**)

**Supplementary Table 4. Process evaluation components for the Feel Good Study**

| Component | Question |
| --- | --- |
| Recruitment | 1. What procedures were followed to recruit schools, children and families? |
| Fidelity | 1. To what extent was each of the study components^*^ implemented as planned. |
| Dose delivered | 1. To what extent were all study components^*^ delivered? |
| Dose received | 1. To what extent did children, family and schools/teachers enjoy the Feel Good study? 2. Were the researchers and schools satisfied with the Feel Good study? |
| Reach | 1. What % of the children within a classroom were the Feel Good Study lessons delivered to? |
| Context | 1. What were barriers and facilitators to implementing the Feel Good study? |

^*^For the purpose of process evaluation, study components refers to both study processes and intervention elements.

### Process evaluation instruments

Consideration of program resources, characteristics and context is an important step in process evaluation plan development. Tools that were realistic within resource availability and timeline of the Feel Good Study were check-lists, peer-review forms, brief satisfaction scales, feedback surveys, and other analytics (e.g., Facebook analytics). Given time constraints, the tools were not pilot-tested prior to actual implementation, but were reviewed by researchers and target users. Minor modifications were made to instruments designed for the wait-list control group to ensure relevancy, whilst remaining similarity for comparisons. A summary of data sources used to support specific process evaluation components of the Feel Good Study is provided in **Supplementary table 5**.

A ‘core components’ checklist was designed as the primary measure to evaluate intervention fidelity and dose delivered, and was completed and cross-checked at the end of intervention by two researchers (NG and JR). The checklist included 18 items for the intervention group, which were adapted to 13 items relevant for the control group (see **Supplementary table 6** for details of included items). Each item was scored on a 5-point Likert scale from strongly agree to strongly disagree. For the purpose of evaluating fidelity, categorical responses were converted to a continuous scale from 1 (strongly disagree) to 5 (strongly agree). Researchers also completed observation checklists and peer-review forms throughout the intervention as part of formative data collection.

Teachers and parents/caregivers completed an end of intervention survey, which included both quantitative and optional qualitative feedback. Qualitative data was collected on 5-point Likert scales, ranging from “Very satisfied” or “Strongly Agree” to “Very dissatisfied” or “Strongly disagree”, and including “not applicable to me” and “prefer not to answer” options. Children completed brief 5-point hedonic satisfaction and enjoyment scales at the end of each lesson in surveys that were integrated into their lesson workbooks. This data was primarily used to assess dose received. For ease of categorical data presentation, strongly positive/positive and strongly negative/negative response categories have been collapsed.

Facebook analytics were exported at the end of the intervention for summative assessment, with key metrics including number of page views, post clicks, reactions, comments and shares, and total video views and viewing behaviour details. Facebook analytics were informally monitored on a weekly basis as part of formative process evaluation, for example if videos were attracting engagement at a certain time, then future video posts would be prioritised for that time. This data gives supporting evidence for dose received and reach, as it provide insights into participant satisfaction and the extent to which all methods/activities were used.

Text messages were sent weekly to parents/caregivers receiving the vegetable boxes. Parents/caregivers were asked if they had received their vegetable box (Yes/No), and if they had any feedback (e.g., quality issues), which was used as summative and formative data, respectively.

## Process evaluation components

**Supplementary Table 5. Final evaluation plan methods for the Feel Good study.**

| Process evaluation component | Process evaluation question | Data sources | Tools/procedures | Time of data collection |
| --- | --- | --- | --- | --- |
| Recruitment | 1. What procedures were followed to recruit schools, children and whānau (parents/caregivers)? | - Researchers | Researchers document all recruitment activities, including barriers/facilitators; as well as informal feedback in field notes | Researchers report throughout recruitment period. |
| Fidelity (quality) | 1. To what extent was each of the intervention elements implemented as planned? | - Researchers - Teachers - Parent/caregiver | *Primary measure:*   - Core component checklist (See supplementary Table 5) | Researchers report at end of intervention |
|  |  |  | *Supporting measures:*   - Lesson observation checklist - Peer-review forms - Vegetable box text message^1^ - Teacher survey^2^ - Parent survey^2^ | Researchers report throughout intervention  Parent/teacher report at end of intervention |
| Dose delivered (completeness) | 1. To what extent were all intervention components delivered? | - Researchers - Teachers - Parent/caregiver | *Primary measure:*   - Core component checklist | Researchers report at end of intervention |
|  |  |  | *Supporting measures:*   - Vegetable box text message - Lesson observation checklist - Peer-review forms - Teacher survey - Parent survey - Facebook analytics review | Researchers report throughout intervention  Parent/teacher report at end of intervention |
| Dose received (satisfaction) | 1. To what extent did children, whānau (parents/caregivers) and teachers enjoy the Feel Good study intervention? 2. Were the researchers and schools satisfied with the Feel Good study intervention? | - Researchers - Teachers - Parent/caregiver - Children | *Primary measures:*   - Brief satisfaction scales in children’s workbook - Vegetable box text message - Teacher survey - Parent survey | Children report end of each lesson  Parent & teacher report end of intervention |
|  |  |  | *Supporting measures:*   - Lesson observation checklist - Peer-review forms - Teacher survey - Parent survey - Facebook analytics review | Researchers report throughout intervention  Parent/teacher report end of intervention |
| Reach (participation rate) | 1. What % of the children within a classroom were the Feel Good study lessons delivered to? | - Researchers | - Classroom attendance register | Taken for each class in which the lessons were taught |
| Context | 1. What were barriers and facilitators to implementing the Feel Good study? | - Researchers - Teachers - Parent/caregiver | - Researchers document barriers and facilitators in informal field notes during study. - Teacher survey - Parent survey | Administered at end of intervention |

**^1^**Each week, participants received a text message asking if they had received their vegetable box (Yes/No), and if they had any feedback they wanted to pass on (e.g., quality issues). ^2^

**Supplementary Table 6. Core components checklist item descriptions for intervention and control groups^1^**

|  | **Intervention group** | **Control group** |
| --- | --- | --- |
| **School support package** | Box of fruit delivered to classroom weekly | NA |
|  | Researchers delivered 1x primer lessons as planned | NA |
|  | Researchers delivered 5x sensory lessons as planned | Researchers delivered 1x sensory lesson as planned |
|  | Researchers provided the workbook and support to complete the activities | Researchers provided the workbook and support to complete the activities at home |
|  | Researchers provided reward stickers at the end of each lesson | Researchers provided reward stickers at the end of the lesson |
|  | Children grew a portable garden together | Children grew a portable garden together |
| **Home support package** | Box of vegetables delivered participants household weekly | Box of vegetables and fruit delivered participants household weekly |
|  | Researchers provided families with printed resources | Researches provided families with printable resources |
|  | Researchers invited parents/caregivers to join the private Facebook group | NA |
|  | Researchers provided inspirational videos and posts via Facebook | Researchers provided inspirational videos and posts via email |
|  | Researchers answered queries via Facebook and commented on community posts | NA |
|  | Researchers sent weekly emails sharing a summary of information posted on Facebook | NA |
|  | Researchers provided opportunities for parent/caregiver feedback | Researchers provided opportunities for parent/caregiver feedback |
| **Study processes** | Researchers offered a face-to-face welcome meeting to parents/caregivers | Researchers offered a face-to-face welcome meeting to parents/caregivers |
|  | Researchers completed data collection at study visits | Researchers completed data collection at study visits |
|  | Researchers introduced children to the NIH toolbox and veggie meter before conducting testing | Researchers introduced children to the NIH toolbox and veggie meter before conducting testing |
|  | Researchers provided all questionnaires to parents/caregivers via REDCap | Researchers provided all questionnaires to parents/caregivers via REDCap |
|  | Researchers provided instructions to parents/caregivers on stool sample collection | Researchers provided instructions to parents/caregivers on stool sample collection |

^1^The core components checklist was completed and then cross-checked by researchers at the end of the intervention, and was primarily designed to evaluate dose delivered and fidelity components of the process evaluation relevant to both study procedures (e.g., data collection) and intervention elements. Researchers completed the core components checklist for each participating school on a 5-point scale from strongly agree to strongly disagree.

### Stool sample collection methodology

The study aimed to have a random sample of 10 children to complete a faecal sample collection at baseline and the 10-week follow-up. This was an entirely optional measurement, and required additional child assent and parent/caregiver consent. Children’s stool samples were collected in the 24-48h prior to scheduled baseline and follow-up data collection timepoints using the OMNIgene.GUT home collection kits. Parents sent samples directly to researchers at the University of Auckland via biological sample couriers. Samples were then discarded, since further analysis was not required for the primary objective of assessing collection feasibility in this study.

# Supplementary results

**Supplementary Table 7. Summary of data collection rates at baseline and follow-up.**

|  | Time | Total | Intervention | | Control | |
| --- | --- | --- | --- | --- | --- | --- |
|  |  |  | High SES | Low SES | High SES | Low SES |
| **Enrolled** |  | 70 | 22 | 16 | 20 | 12 |
| **Children’s data** |  |  |  |  |  |  |
| Questionnaires – diet, positive affect | Baseline | 68 (97) | 22 (100) | 15 (94) | 19 (95) | 12 (100) |
|  | Follow-up | 63 (90) | 22 (100) | 13 (81) | 17 (85) | 11 (92) |
| Veggie Meter | Baseline | 66 (94) | 22 (100) | 14 (88) | 19 (95) | 11 (92) |
|  | Follow-up | 61 (87) | 22 (100) | 12 (75) | 17 (85) | 10 (83) |
| NIH toolbox | Baseline | 66 (94) | 22 (100) | 14 (88) | 19 (95) | 11 (92) |
|  | Follow-up | 61 (87) | 22 (100) | 12 (75) | 17 (85) | 10 (83) |
| **Parents/caregivers data** |  |  |  |  |  |  |
| Questionnaires – diet, behaviour | Baseline | 65 (93) | 20 (91) | 15 (94) | 19 (95) | 11 (92) |
|  | Follow-up | 60 (86) | 19 (86) | 14 (88) | 17 (85) | 10 (83) |

Values presented as n (%), referring to the proportion of enrolled participants who completed data collection.

**Supplementary Table 8. Summary of biological sample collection feasibility**

|  |  | Intervention | | Control | |
| --- | --- | --- | --- | --- | --- |
|  | Total | High SES | Low SES | High SES | Low SES |
| **Enrolled** | 70 | 22 | 16 | 20 | 12 |
| **Recruitment** |  |  |  |  |  |
| Child assent | 19 (27) | 5 (23) | 4 (25) | 9 (45) | 1 (8) |
| Parent consent | 33 (47) | 9 (41) | 7 (44) | 12 (60) | 5 (42) |
| Full consent (parent/child) | 17 (24) | 3 (14) | 4 (25) | 9 (45) | 1 (8) |
| **Collection** |  |  |  |  |  |
| Participation^1^ | 9 | 2 | 1 | 5 | 1 |
| Baseline collected | 4 (44) | 1 (50) | 0 (0) | 2 (40) | 1 (100) |
| Follow-up collected | 2 (22) | 1 (50) | 0 (0) | 0 (0) | 1 (100) |

Values presented as n (%), referring to the proportion of enrolled participants (recruitment) or of those participants taking part in biological sample collection (collection). ^1^ Randomly selected based on participants who had fully consented for stool sample collection (i.e., both parent consent and child assent) before first day of data collection. Because less than the target of 10 participants had consented in time, all 9 participants consenting were invited to participate in biological sample collection.

**Supplementary table 9. Summary of Feel Good Study implementation (dose delivered and fidelity) for intervention and control groups.**

| Intervention component | Intervention group | | | Control group | | |
| --- | --- | --- | --- | --- | --- | --- |
|  | School A | School B | Mean fidelity rating ^1^ | School C | School D | Mean fidelity rating ^1^ |
| School-based elements |  |  |  |  |  |  |
| Fruit box delivery | ✓ | ✓ | 80% | - | - | - |
| Primer Lesson | ✓ | ✓ | 60% | - | - | - |
| Sensory lessons | ✓ | ✓ | 100% | ✓ | ✓ | 100% |
| Study workbook and activities | ✓ | ✓ | 80% | ✓ | ✓ | 80% |
| Reward stickers | ✓ | ✓ | 80% | ✓ | ✓ | 100% |
| Portable vegetable garden | ✓ | ✓ | 90% | ✓ | ✓ | 100% |
| *Mean fidelity of school-based elements* |  |  | **82%** |  |  | **95%** |
| Home-based elements |  |  |  |  |  |  |
| Vegetable box delivery | ✓ | ✓ | 100% | ✓ | ✓ | 100% |
| Printed or printable recipe resources provided | ✓ | ✓ | 40% | ✓ | ✓ | 100% |
| Facebook group invitations offered | ✓ | ✓ | 100% | - | - | - |
| Regular tips & tricks posted to Facebook and/or email | ✓ | ✓ | 100% | ✓ | ✓ | 100% |
| *Mean fidelity of home-based elements* |  |  | **85%** |  |  | **100%** |
| Study processes |  |  |  |  |  |  |
| Face-to-face welcome meeting to parents | ✓ | ✓ | 100% | ✓ | ✓ | 100% |
| Data collection completed at study visits | ✓ | ✓ | 100% | ✓ | ✓ | 100% |
| Children introduced to data collection tools before testing | ✓ | ✓ | 100% | ✓ | ✓ | 100% |
| Questionnaires provided to parents via REDCap | ✓ | ✓ | 100% | ✓ | ✓ | 100% |
| Parents provided with instructions for stool sample collection | ✓ | ✓ | 100% | ✓ | ✓ | 100% |
| *Mean fidelity of study processes* |  |  | **100%** |  |  | **100%** |

^1^Categorical responses for each item were converted to a continuous scale from 1 (strongly disagree) to 5 (strongly agree) to evaluate intervention fidelity (the extent to which the intervention was implemented as planned). Data is presented as average % of the total score from the two intervention schools and two control schools, with 100% representing implementation completely as planned.

**Supplementary Table 10. Process Evaluation summary – Child report at end of each sensory experiential learning lesson**.

|  | A lot like you/ Mostly like you | Like you | Sometimes like you/Not like you |
| --- | --- | --- | --- |
|  | 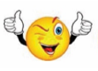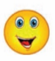 | 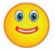 | 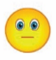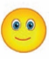 |
| Intervention group - Primer lesson (n=33) |  |  |  |
| Had fun in lesson | 29 (88) | 3 (9) | 1 (3) |
| Enjoyed activity 1 (portable garden) | 30 (93) | 0 (0) | 1 (3) |
| Intervention group - Lesson 1 (Sight, n=36) |  |  |  |
| Had fun in lesson | 36 (100) | 0 (0) | 0 (0) |
| Enjoyed activity 1 (perfect v ugly produce) | 31 (86) | 3 (8) | 0 (0) |
| Enjoyed activity 2 (blind taste test) | 29 (88) | 3 (8) | 1 (3) |
| Enjoyed activity 3 (monster design) | 30 (93) | 3 (8) | 1 (3) |
| Intervention group - Lesson 2 (Smell, n=34) |  |  |  |
| Had fun in lesson | 32 (94) | 1 (3) | 1 (3) |
| Enjoyed activity 1 (mystery smell jars) | 32 (94) | 1 (3) | 1 (3) |
| Enjoyed activity 2 (smell contest) | 31 (91) | 2 (6) | 1 (3) |
| Intervention group - Lesson 3 (Touch, n=24) |  |  |  |
| Had fun in lesson | 23 (96) | 1 (4) | 0 (0) |
| Enjoyed activity 1 (explore table) | 23 (96) | 1 (4) | 0 (0) |
| Intervention group - Lesson 4 (Sound, n=28) |  |  |  |
| Had fun in lesson | 25 (89) | 2 (7) | 1 (4) |
| Enjoyed activity 1 (explore table) | 25 (89) | 3 (11) | 0 (0) |
| Enjoyed activity 2 (fruit and vegetable bugs) | 25 (89) | 2 (7) | 1 (4) |
| Intervention group - Lesson 5 (Taste, n=35) |  |  |  |
| Had fun in lesson | 32 (91) | 2 (6) | 1 (3) |
| Enjoyed activity 1 (explore table) | 30 (86) | 4 (11) | 1 (3) |
| Enjoyed activity 2 (flavour combinations) | 25 (71) | 4 (11) | 6 (17) |
| Control group – “Mega Lesson” (n=25). |  |  |  |
| Had fun in lesson | 25 (100) | 0 (0) | 0 (0) |
| Enjoyed activity 1 (portable garden) | 23 (92) | 0 (0) | 2 (8) |
| Enjoyed activity 2 (fruit and vegetable bugs) | 23 (92) | 1 (4) | 1 (4) |

Data is presented as n (%). There were minimal “did not answer” responses, ranging from 6-8% for intervention group primer lesson and lesson 1.

**Supplementary Table 11.** Summary of behaviour change uptake according to parent’s end of intervention survey.

|  | Intervention group  (n=32, 84% response rate) | Control group  (n=21, 66% response rate) |
| --- | --- | --- |
| Eating the vegetable box together as a family | 78% | 90% |
| Talking about fruits and vegetables with children | 81% | 81% |
| Cooking meals together as a family | 63% | 67% |
| Reducing food waste | 66% | 48% |
| Using recipes provided by the study | 53% | 29% |
| Gardening together as a family | 25% | 33% |

Data is presented as proportion of respondents that had tried each item as a result of taking part in the study.

**Supplementary Table 12. Teacher-reported feedback in end of intervention survey.**

|  | Intervention group, n=2 (100% response rate) | | | Control group, n=2 (100% response rate) | | |
| --- | --- | --- | --- | --- | --- | --- |
|  | Very satisfied/satisfied | Neutral | Very dissatisfied/  dissatisfied | Very satisfied/satisfied | Neutral | Very dissatisfied/  dissatisfied |
| **Intervention components** |  |  |  |  |  |  |
| Delivery of sensory lessons to classroom | 2 (100) | - | - | 1 (100)^1^ | - | - |
| Fruit box - Quality | 2 (100) | - | - | 2 (100)^2^ | - | - |
| Fruit Box - Variety | 2 (100) | - | - | 2 (100) | - | - |
| Fruit Box - Amount | 2 (100) | - | - | 2 (100) | - | - |
| Facebook group | 2 (100) | - | - | 2 (100) | - | - |
| Portable vegetable garden | 2 (100) | - | - | 2 (100) | - | - |
| Receiving the fruit has had a positive impact on my class | 2 (100) | - | - | 2 (100) | - | - |
| I would be willing to teach the sensory lessons to my class | 2 (100) | - | - | 2 (100) | - | - |
| **Study participation** |  |  |  |  |  |  |
| My class enjoyed taking part in the study | 2 (100) | - | - | 2 (100) | - | - |
| I would recommend other schools to take part in the study | 2 (100) | - | - | 2 (100) | - | - |
| **Research processes** |  |  |  |  |  |  |
| It was easy to take part in the study | 2 (100) | - | - | 2 (100) | - | - |
| It was easy to talk with and contact the researchers | 2 (100) | - | - | 2 (100) | - | - |
| I was well informed about what taking part would involve | 2 (100) | - | - | 2 (100) | - | - |

Data is presented as n (%). ^1^One teacher was not present for the sensory lesson delivery, and could not respond to this question. ^2^Control groups received a one-off fruit box at the end of data collection.

**Supplementary Table 13**: **Summary of qualitative feedback received by the parent/caregiver and teacher online feedback surveys.**

| Theme | Illustrative quotes in response to ““What did you enjoy most about the study?” and “Is there anything else that you would like to tell us?” |
| --- | --- |
| Promoting children’s food acceptance | **Parents/caregivers^1^:**  "This study made my son take interest in gardening. He often helps in vegetable gardening."  "My child's curiosity about fruits and veggies"  "Trying things different fruits and veges in class was great as it made him more receptive to trying something new :)"  "My daughter cooked way more and tried way more variety of veges"  "Encouraged my child to try a broader range of fruit and vegs"  "We've learned how to make a lot of recipe using the fruits and vegetables given."  "That my child is more open to eating a variety of vegetables, and willing to try new ones."  "The vege box had some items we don't normally buy which was a great opportunity for my girls to try new things."  **Teachers:**  “It was a great opportunity for the children to be more adventurous with fruit, they often said that they tried things they wouldn't normally have had, or things they'd never tried before. I loved the portable garden idea, and it's something I'll look to do in future with other students.”  “The researchers were knowledgeable, organised, friendly and fun; kids loved trying new fruit and vegetables and doing the sensory experiences! Lots of happy faces and fun tied into the eating of fruit and veg, which is a great connection for kids to have to form those healthy habits.” |
| Access and availability of fruit and vegetables | **Parents/caregivers^2^:**  "Getting veg boxes and talking about it with our son"  "[Child name] learned to cut veggies at school and she started eating veggies at home as salad."  "The encouragement it gave us to include more variety of fruit and vegetables in our diet. It helped us to get out of a 'rut' with our own selections"  "I appreciated the free veggie boxes and it prompted me to cook new and different meals that I usually would. It encouraged me to try to eat more veggies." |
| Gratitude | **Parents/caregivers^3^:**  "Great initiative, keep it up."  "I would really be keen to continue receiving the boxes, perhaps a future venture to buy these after the tests could be considered in future."  "We are just very grateful we could participate in the study."  "Thank you for promoting awareness and healthy eating habits among children."  **Teachers:**  “Thank you for the experience. We are very grateful for the fruit and vegetables we received. It certainly uplifted attitudinal changes in cooking, eating more fruit and vegetables and generally felt lovely to be given something in return, especially with the changing climate in society with all of the negative aspects being highlighted day to day. A big thank you to the researchers for their mahi and the respect and care they put into everything they did. We enjoyed meeting you all. Na mihi nui”  “Fantastic study, to see if eating more fruits and vegetables could improve children's mental and brain health, enhancing their cognitive and emotional abilities. Fantastic opportunity.”  Just that it was an amazing opportunity and I'm so pleased that we were able to take part. |

Anonymisation and minor adjustments to spelling and grammar/punctuation were made to improve readability without changing the original meaning. Some response not presented to avoid reception including comments on ^1^The opportunity to try a wide variety of produce (n=9), free produce box deliveries (n=2), and ^2^Including a variety of vegetables (n=3), encouraging healthy eating (n=2); ^3^ enjoyment of being part of the study (n=5).

**Supplementary Table 14**: **Summary of qualitative feedback received by the parent/caregiver and teacher online feedback surveys in response to the question “What are some things that you would like to see changed?” and any additional feedback received.**

| Feedback source | Illustrative Quotes |
| --- | --- |
| “What are some things that you would like to see changed?” | **Parents/caregivers^1^:**  “Student should spend more time in this type of study.”  “More parent involvement for more inspiration (recipes)”  “next time there is a study it would be good to have some fruit as well”  “Friday late afternoon is not a good time for delivery.”  “More tips on how to encourage picky children”  “I don't belong to FB and have lost my login, which I did not want to reset all my other passwords  Could have been  accepted into the group as I wasn't part of any FB discussions”  “Information in the box about what the veges were - some weeks i didn't know.  More recipes information in the box and on FB”  **Teachers:**  “Nil (We loved the whole process). However, we were disappointed that we didn't get chosen as the main group. We would have liked to have been apart of the whole project.”  “The variety of the fruit was an issue, there was a glut of bananas which they weren't keen on.”  “I'd just say at year 6, maybe they even use the kitchen facilities at schools to try recipes out on certain days that are planned in advance.” “They could even each plant different things to be used in a big group recipe at the end of the study. A cookbook complied by the student work would've been fun, too. I had that idea and gave homework on using the veg they received for recipes to compile- just didn't have time.”  “Just maybe less kiwi fruit in the boxes. 😊 There was a massive amount! I just felt bad as it seemed like so many, so we tried to eat them and give them out as much as possible, but so many that kids stopped wanting to take them home towards the end. It was quite awesome to have so much fruit in the room, though!! They helped themselves whenever they wanted fruit, and it was such a novelty. They loved their fruit buffet! ❤️🍎🍊🍌 Thank you!” |
| Feedback received via email/text message regarding vegetable deliveries during the 10-week intervention period. | **Parents/caregivers^2^:**  “Sadly I can’t say quality of veg is good. Cucumber is so soft on one side and radish rotten on the base. Not sure if those people check quality of vegetables before sending.”  “The veggie delivery just came and the quality looks much fresher. Excited to try some new things with my kids.”  “Box just arrived and it’s great. Produce is really fresh and in good condition (email feedback). The box arrived Friday and it was well packaged and really fresh and lovely. Thank you. (txt feedback)” |
| Other teacher feedback: | “The researchers were flexible and had a genuine interest in the well-being of the students and teacher. They were approachable and extremely flexible with the needs of the school teaching day. We loved having our fruit and vegetable readings. This motivated us all to increase our fruit and vegetable intake.”  “[I enjoyed most the] interactive classroom sessions and learning through the five senses being hands on.” |

Anonymisation and minor adjustments to spelling and grammar/punctuation were made to improve readability without changing the original meaning. Some response not presented to avoid reception including comments on ^1^Recipe variety (n=3) and including fruit in the produce boxes provided (n=1), and ^2^Good vegetable quality (n=2), poor vegetable quality (n=1).

## Process evaluation (Context) – barriers/facilitators to implementation.

From researcher observations and end of intervention surveys, the key barriers for intervention implementation centred on timing of intervention delivery and quality of produce boxes, while the most important facilitator was maintaining good relationships with schools and teachers.

**Timing of delivery -** Conducting the intervention in Term 4 (last term of the school year) was challenging due to various end of year activities (e.g., school camp, athletics, graduation), resulting in student absences and class disruptions. Additionally, it was not possible to deliver the wait-list control until the following year when students have moved to different classrooms.

**Quality of fresh produce boxes** – Over 90% of parents in the study were either very satisfied or satisfied with the quality of the produce, and feedback was generally positive. However, 9% of intervention group responded neutrally and 5% of the control group were dissatisfied with the quality of the vegetable box.

**Relationships with schools and teachers -** From researcher observations, proactive and enthusiastic teachers resulted in higher recruitment rates and engagement during the sessions. Other observed facilitators included highlighting benefits of taking part in the research (e.g. supporting children’s mental health and diet), offering incentives/rewards (i.e. school voucher, lessons delivered by researchers, prize for the meal photo challenge), incorporating responsive teaching practices alongside cultural considerations (e.g. integrating karakia blessing) that is familiar to the classroom, and tailoring language and content according to student needs.

## Formative process evaluation measures

The average response rate of participants that responded to the weekly SMS message veggie box delivery check-ins was 75%. All of these participants replied “Yes” they did receive their vegetable box; it was assumed that non-responders had also received their box.


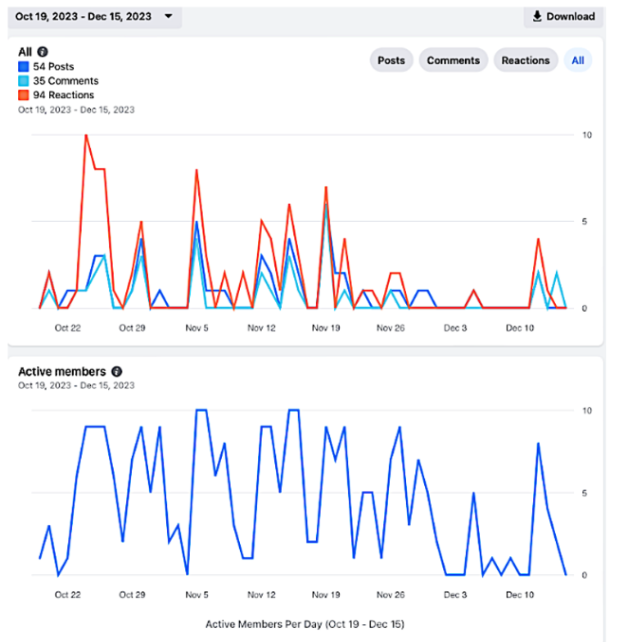

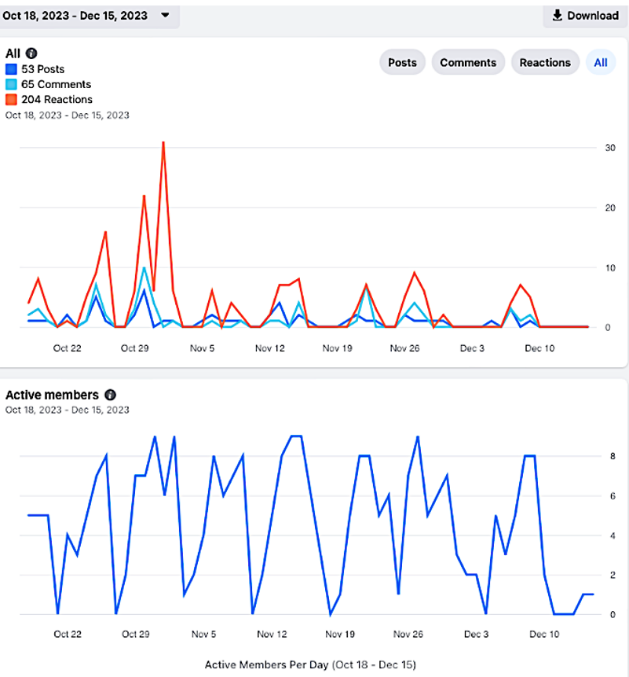


**Supplementary Figure 2. Facebook analytics showing member engagement during the intervention period.**

# References

1. Michie S, van Stralen M, West R. The behaviour change wheel: a new method for characterising and designing behaviour change interventions. Implement Sci 2011;6:1–12.

2. Michie S, Ashford S, Sniehotta FF, Stephan U, Bishop A, French DP, et al. A refined taxonomy of behaviour change techniques to help people change their physical activity and healthy eating behaviours : The CALO- RE taxonomy. 2011;0446.

3. Blanchette L, Brug J. Determinants of fruit and vegetable consumption among 6-12-year-old children and effective interventions to increase consumption. J Hum Nutr Diet 2005;18:431–43.

4. Evans CEL, Christian MS, Cleghorn CL, Greenwood DC, Cade JE. Systematic review and meta-analysis of school-based interventions to improve daily fruit and vegetable intake in children aged 5 to 12 y. Am J Clin Nutr 2012;96:889–901.

5. Pearson N, Biddle SJH, Gorely T. Family correlates of fruit and vegetable consumption in children and adolescents: A systematic review. Public Health Nutr 2009;12:267–83.

6. Gerritsen S, Harre S, Rees D, Renker-Darby A, Bartos A, Waterlander W. Community group model building as a method for engaging participants and mobilising action in public health. Int J Environ Res Public Health 2020;17:3457.

7. Wardle J, Huon G. An experimental investigation of the influence of health information on children’s taste preferences. 2000;15:39–44.

8. Poelman AAM, Cochet-Broch M, Cox DN, Vogrig D. VERTICAL: A Sensory Education Program for Australian Primary Schools to Promote Children’s Vegetable Consumption. J Nutr Educ Behav 2017;49:527-528.e1.

9. Battjes-fries M, Haveman-Nies A, Zeinstra G, van Dongen E, Meester H, R van den T-P, et al. Effectiveness of Taste Lessons with and without additional experiential learning activities on children’s willingness to taste vegetables. Appetite 2017;109:201–8.

10. Mustonen S, Tuorila H. Sensory education decreases food neophobia score and encourages trying unfamiliar foods in 8 – 12-year-old children. Food Qual Prefer Elsevier Ltd; 2010;21:353–60.

11. Reverdy C, Chesnel F, Schlich P, Koster E, Lange C. Effect of sensory education on willingness to taste novel food in children. Appetite 2008;51:156–65.

12. Laureati M, Bergamaschi V, Pagliarini E. School-based intervention with children. Peer-modeling, reward and repeated exposure reduce food neophobia and increase liking of fruits and vegetables. Appetite 2014;83:26‐32.

13. Saunders RP, Evans MH, Joshi P. Developing a Process-Evaluation Plan for Assessing Health Promotion Program Implementation: A How-To Guide. Health Promot Pract 2005;6:134–47.
